# Case of Disease of the Testicle

**Published:** 1826-09

**Authors:** 

**Affiliations:** St. George's Hospital


### DISEASE OF THE TESTICLE.

*Case of Disease of the Testicle.* Treated at St. George's Hospital, by Mr. Brodie.

Henry Line, twenty-six years of age, admitted into St. George's Hospital, July 12th, 1826.—The left testicle was enlarged to the size of a goose's egg, and of an irregular oval form. The tumor was ponderous, and for the most part hard to the touch; but in some parts there was an indistinct feeling of fluctuation. There was neither pain nor tenderness in the testicle itself; but there was a slight pain in the back.

The patient stated, that the enlargement of the testicle had begun three months ago, without pain; and that it had gradually increased, still without pain in the part itself, although he soon began to experience a slight degree of pain in the loins.

In order to ascertain whether there was really fluid collected in those parts, in which there was an apparent fluctuation, a puncture was made with a narrow sharp-pointed instrument; but no fluid was discharged.

Mr. Brodie was led to believe, from the history and symptoms, that the tumor was of such a nature as did not admit of relief, except from an operation. However, it was thought prudent to put the patient under the influence of mercury before an operation was finally determined on; and accordingly the following pills were prescribed:

Calomelanos gr. ij.; Pulv. Antimon. gr. j.; Opii pulv. gr. ss. fiat pilula ter die sumenda.

—These pills were taken for about a fortnight, and the gums were made sore; but there was no diminution of the size of the tumor, nor any relief from the pain in the loins.

August 4th.—The testicle was removed in the usual manner, the vessels being secured by separate ligatures, after the division of the chord. Two or three days after the operation, there were symptoms indicating an inflammatory affection of the chest; but these yielded to the usual remedies, and at present (August 13th,) the patient is going on quite well, and the wound is healing.

On making a section of the testicle after the operation, the diseased structure was found to consist of two parts: first, a multitude of small hydatid-like cysts, containing serum, varying from the size of a seed of millet to that of a large pea; secondly, a grey firm substance, somewhat of a ligamentous structure, in which the hydatid-like cysts were imbedded, and to which they had a loose adhesion. It appeared, on the first inspection, as if the whole of the testicle had been converted into this diseased mass; but, on more minute examination, it was ascertained that the glandular structure of the testicle was entire, and in a perfectly healthy state, occupying every where the circumference of the tumor, and forming a thin layer on its surface. The epididymis was also in a perfect state at the posterior part of the testicle, and the spermatic cord was free from disease. There were no preternatural adhesions of the tunica vaginalis, and nothing more than the usual moisture on its surface.

---

